# Biophysical Properties and Motility of Human Mature Dendritic Cells Deteriorated by Vascular Endothelial Growth Factor through Cytoskeleton Remodeling

**DOI:** 10.3390/ijms17111756

**Published:** 2016-10-31

**Authors:** Zu-Quan Hu, Hui Xue, Jin-Hua Long, Yun Wang, Yi Jia, Wei Qiu, Jing Zhou, Zong-Yao Wen, Wei-Juan Yao, Zhu Zeng

**Affiliations:** 1 Key Laboratory of Biological and Medical Engineering, Guizhou Medical University, Guiyang 550025, China; huzuquan@gmc.edu.cn (Z.-Q.H.); xuehui@bjmu.edu.cn (H.X.); wangyun@gmc.edu.cn (Y.W.); jiayiyouxiang@163.com (Y.J.); qwei407@163.com (W.Q.); zhou323jing126@126.com (J.Z.); 2 Engineering Research Center of Medical Biotechnology, Guizhou Medical University, Guiyang 550025, China; 3 School of Biology and Engineering, Guizhou Medical University, Guiyang 550025, China; 4 Department of Head and Neck, Affiliated Cancer Hospital, Guizhou Medical University, Guiyang 550025, China; longjinhua100@sina.cn; 5 Hemorheology Center, School of Basic Medical Sciences, Health Science Center of Peking University, Beijing 100191, China; rheol@bjmu.edu.cn

**Keywords:** dendritic cells, vascular endothelial growth factor, biophysical characteristics, motility, immune function

## Abstract

Dendritic cells (DCs), the most potent antigen-presenting cells, play a central role in the initiation, regulation, and maintenance of the immune responses. Vascular endothelial growth factor (VEGF) is one of the important cytokines in the tumor microenvironment (TME) and can inhibit the differentiation and functional maturation of DCs. To elucidate the potential mechanisms of DC dysfunction induced by VEGF, the effects of VEGF on the biophysical characteristics and motility of human mature DCs (mDCs) were investigated. The results showed that VEGF had a negative influence on the biophysical properties, including electrophoretic mobility, osmotic fragility, viscoelasticity, and transmigration. Further cytoskeleton structure analysis by confocal microscope and gene expression profile analyses by gene microarray and real-time PCR indicated that the abnormal remodeling of F-actin cytoskeleton may be the main reason for the deterioration of biophysical properties, motility, and stimulatory capability of VEGF-treated mDCs. This is significant for understanding the biological behavior of DCs and the immune escape mechanism of tumors. Simultaneously, the therapeutic efficacies may be improved by blocking the signaling pathway of VEGF in an appropriate manner before the deployment of DC-based vaccinations against tumors.

## 1. Introduction

Dendritic cells (DCs) are the most potent and specialized antigen-presenting cells and essential for the initiation of immune responses. They can be divided into immature and mature types through their functional status. Immature DCs (imDCs) reside in peripheral tissues and specifically capture and process foreign antigens. Then, they gradually differentiate to mature DCs (mDCs) and migrate to secondary lymph nodes, physically interacting with naive T cells to initiate immune responses. In this process, the expression of co-stimulatory molecules is upregulated; the antigen-presenting capacity is enhanced; and chemokine receptors are expressed for promoting migration [1]. A number of diseases have been attributed to the deteriorated functions of DCs, including the differentiation, maturation, and motility of DCs, and their capacities of antigen capture, processing, and presentation. Thus, DC-targeted or -based immunotherapies have been exploited for the treatment of immune-associated diseases [2,3,4,5,6].

DCs are crucial for the initiation and maintenance of antitumor immune responses. In tumor tissues, DCs can uptake apoptotic or necrotic tumor cells and process tumor-associated antigens. After maturation and migration through lymphatic vessels to the draining lymph nodes, they dynamically interact with T cells and present antigens in the context of cell surface major histocompatibility complex (MHC) class I and II molecules. Then the activated effector T cells emigrate and infiltrate to the tumor tissue for destruction of tumor cells [7,8,9]. The migratory capacities for both DCs and T cells are required for antitumor immunity [10,11,12]. Simultaneously, DCs-based vaccination against cancer has been exploited for clinical application and great progress has been achieved [13,14,15]. Despite this strategy is considered as a promising approach for the immunotherapy of cancer, its therapeutic efficiency is still unsatisfied in clinical trials [14]. One of the primary reasons is their impaired migration capabilities [14,16], inferring that the cell motility play vital roles in its performing normal immune functions and DCs-based immunotherapy against cancer.

The tumor microenvironment (TME) consists of immune cells, stroma cells, and the extracellular matrix, which is a battleground for tumor cells and the immune system during the neoplastic process. It is correlated with the proliferation, survival, metastasis, and immune escape of tumor cells [17]. One of the primary reasons for tumor escape from immune surveillance is that the antigen-presenting cells cannot effectively present tumor antigens and activate naive T lymphocytes, which is caused by TME, not the loss of immunogenicity [18,19,20,21,22]. The immunosuppressive TME can produce many cytokines, including VEGF, interleukin-6 (IL-6), interleukin-10 (IL-10), transforming growth factor-β_1_ (TGF-β_1_), macrophage colony-stimulating factor (M-CSF), nitric-oxide synthase 2 (NOS2), arginase, cyclooxygenase-2 (COX2), indoleamine-2,3-deoxygenase (IDO), prostaglandin E_2_ (PGE_2_), and ganglioside, which solely or cooperatively affect the recruitment, differentiation, maturation, migration, and survival of DCs [17,21,23]. Moreover, the defects of DCs in patients with cancer are systemic and are not localized to tumor tissues [7]. Thus, the TME can inhibit the functions of antigen presentation and T cell activation, resulting in immune nonresponse or immune tolerance to tumor antigens. Biophysical properties of cells can reflect the relationship between structure and function [24,25,26]. The changes of cell biophysical characteristics can directly affect the cell’s deformability, adherence, migration, and interactions with the extracellular matrix [12,27,28,29,30,31]. For the effects of TME on DCs in cancer, the impairment of biophysical properties may be one of the crucial aspects for the inhibition of motility and immune function [20,29]. VEGF, TGF-β_1_, and IL-10 are the most important of suppressive cytokines in TME, and further research should be concentrated on the effects of a single cytokine on DCs, which may be useful for elucidating the potential molecular mechanisms. Our previous study showed that TGF-β_1_ can impair the motility and immune function of mDCs through derangement of biophysical characteristics [21]. VEGF, produced in nearly all tumors, is a key cytokine in tumor development and metastasis. It can promote tumor angiogenesis and inhibit DCs’ differentiation and functional maturation [32,33,34,35,36]. At present, some signaling pathways, such as fms-related tyrosine kinase 1 (Flt-1), nuclear factor kappa-light-chain-enhancer of activated B cells (NF-κB), and signal transducer and activator of transcription 3 (STAT3), have been proven to be involved with the suppression of DCs’ function [34,37,38]. However, it is unclear whether the dysfunction of DCs by VEGF is related to the impairment of biophysical properties and motility.

In this study, the primary objective is to analyze the impact of VEGF on the biophysical characteristics and motility of mDCs, and attempt to explore the potential immune escape mechanisms of tumors. Some interdisciplinary approaches were deployed to characterize the effects of VEGF on mDCs, including electrophoretic mobility (EPM), osmotic fragility, viscoelasticity, transendothelial migration, apoptosis, F-actin cytoskeleton, immune stimulatory capability, and gene expression profiles. The results showed that VEGF impaired the biophysical characteristics of mDCs by remodeling the cytoskeleton, which may be closely associated with the immune function of DCs. The present study provided a new perspective on the immune escape mechanism of tumor cells, indicating that the therapeutic efficiency of DCs-based vaccination may be improved by coupling it with a blocking treatment of the VEGF-associated signaling pathway.

## 2. Results and Discussion

### 2.1. Cell Electrophoretic Mobility

Taking into account that the dose of human serum VEGF was 0.023–1.337 ng/mL and the observed effects on the function of DCs occurred at VEGF serum levels more than 100 times lower than that required in vitro assay, concentrations of 0, 10, 30, 50, and 70 ng/mL were set up to analyze the effects of VEGF on mDCs in vitro, in comparison with previous studies [35,36,37,38] and our preliminary experiments. EPM is an important biophysical parameter that reflects the amounts of negative charges carrying in the cell membrane. Generally, EPM is directly proportional to the negative charges on the cell surface in the same electric field. The cells of eukaryotic organism have negatively charged surfaces in vivo and adhesion of cells to neighboring cells or to organic or inorganic solid surfaces has been suggested, for a long time, to be related to EPM [27,39]. After treatment with different concentrations of VEGF, the effects of VEGF on the surface charge densities of mDCs were determined. The results showed that 50 ng/mL VEGF had the greatest influence on the reduction of EPM of mDCs, followed by 30 ng/mL, 70 ng/mL, and 10 ng/mL (Table 1). T cell priming by DCs occurs in three successive stages: transient serial encounters during the first activation phase are followed by a second phase of long-lasting stable contacts culminating in cytokine production, which makes a transition into a third phase of short contacts and high motility and rapid proliferation of T cells [40]. During the process of antigen presentation, DCs and T cells undergo a direct physical contact in which they must overcome the barrier posed by negatively-charged glycocalyx components [41]. Fewer negative charges on the surface of mDCs could increase the adhesion between DCs and other cells (e.g., naive T cells in lymph node) and/or the extracellular matrix, which might lead to an increased contact time. Previous studies have indicated that the different contact time induces varied immune responses during the interactions between DCs and T cells [40,42,43,44]. Thus, the migration and interaction with T cells of mDCs might be disturbed by VEGF through the decrement of negative charges on cell surfaces, which may be responsible for the immune dysfunction.

### 2.2. Osmotic Fragility

**Figure 1:**
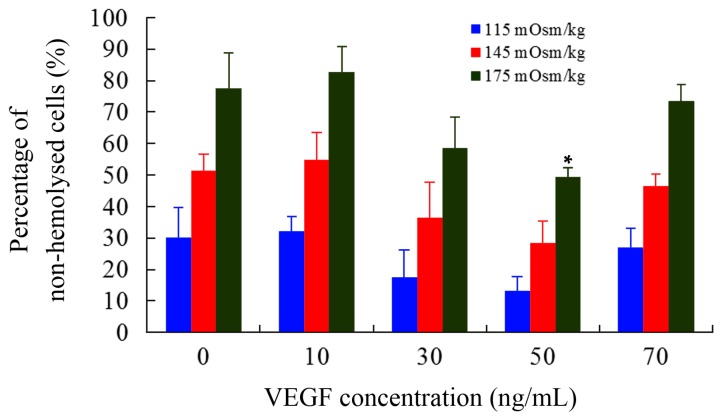
The hemolysis rate of mDCs treated with different concentrations of VEGF. Compared with control group, * *p* < 0.05.

As another important biophysical parameter, the osmotic fragility of cells can reflect their capacities to resist hypo-osmolality. The number of non-hemolyzed mDCs were counted using hemocytometer and the percentage was calculated compared with those of control (295 mOsm/kg). As shown in Figure 1, the percentage was apparently decreased at 50 ng/mL (*p* < 0.05), indicating that mDCs treated with 50 ng/mL VEGF had great decrement of hypo-osmolality resistance. Tumors can damage or disrupt their surrounding tissues or trigger a stress response when supplies are insufficient to meet their oxygen and nutrient demands, which can lead to alternations in local osmotic pressure and metabolic disturbance and generation of reactive oxygen species [45]. Simultaneously, our previous studies revealed that the osmotic fragility of mDCs is significantly lower than those of imDCs, suggesting that imDCs are able to adapt to the different microenvironments at various osmotic concentrations in vivo [41]. It is consistent with their antigen-uptaking function that their podosomes are formed to search for soft spots of low physical resistance in the substrate [12]. Thus, the increased osmotic fragility of mDCs is more sensitive, and the decreased hypo-osmolality resistance of mDCs induced by VEGF cytokine may lead to their cytolysis in TME. This might be one of the reasons for less infiltration of mDCs in tumor tissue and draining lymph nodes and/or for a decline of local immune response in tumors [46,47].

### 2.3. Viscoelasticity Analysis

**Figure 2:**
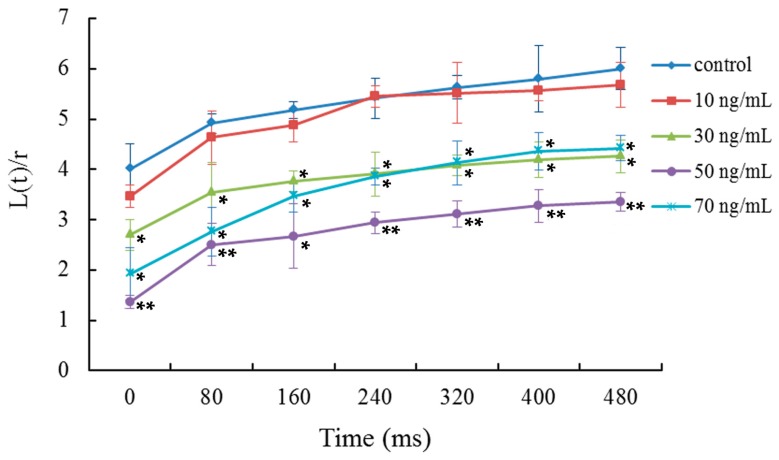
Micropipette aspiration measurements of the viscoelasticity of mDCs treated with different concentrations of VEGF. Relative to the aspiration time, the ratios between the length of cell tongue aspirated into the micropipette, L(t), and the pipette radius, r, were analyzed. Compared with control group, * *p* < 0.05, ** *p* < 0.01.

The viscoelasticity or deformability of mDCs was determined by micropipette measurements. Relative to the aspiration time, the ratios between the length of cell tongue aspirated into the micropipette and the radius of pipette were analyzed (Figure 2). The results indicated that the viscoelastic coefficient of mDCs was significantly decreased when they were treated with 50 ng/mL VEGF, indicating mDCs became less deformable. The time course of cell deformability reflects the mechanical properties of cells and cell interactions. Poor deformability has an adverse influence on cell adhesion with other cells or tissues due to the decrement of contact areas, especially in the presence of shear stress due to blood flow [41]. The results showed that 50 ng/mL VEGF can reduce the deformation capacity of mDCs (Figure 2), indicating that the VEGF-treated mDCs might have trouble attaching to the vascular endothelium or extracellular matrix. Thus, this could affect the subsequent emigration of mDCs from the peripheral tissue or tumor local tissue to draining lymph nodes. In addition, the contact area between mDCs and naive T cells would became smaller as the reduced deformability, which might impair the formation of immune synapses and eventually reduce the activation efficiency of T cells in lymph node. VEGF expression is correlated directly with positive nodal metastases and correlated inversely with the count of mDCs [47], and thus, there are different degrees of influence on mDCs in various tumor patients.

### 2.4. Capacity of Transendothelial Migration

**Figure 3:**
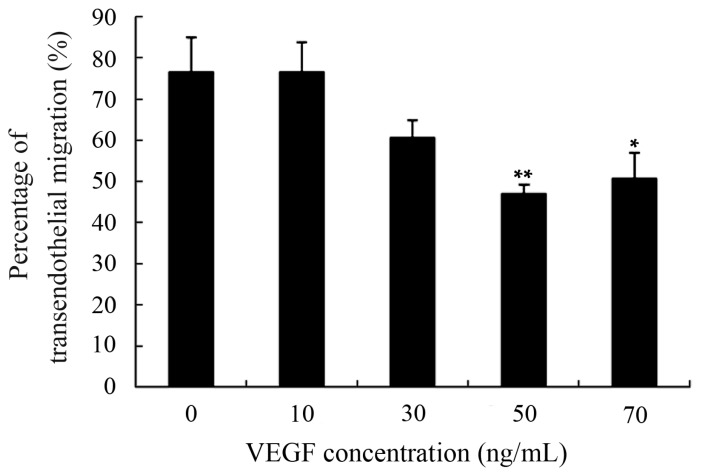
Transwell analysis of the transendothelial migration of mDCs treated with different concentrations of VEGF. The mDCs transmigrating into the lower compartment were counted using a hemocytometer. Compared with control group, * *p* < 0.05, ** *p* < 0.01.

Excellent motility is particularly important for DCs to perform their physiological functions, including antigen-uptaking in peripheral tissues and acquiring antigen presentation in draining lymph nodes [7,8,9]. Moreover, DCs-based vaccines that the DCs loaded with tumor antigens should also have perfect motility to assure that they can migrate from the injection sites to secondary lymphoid tissues and effectively initiate tumor-specific immune responses [14,16]. Transendothelial migration was determined using a Transwell system and the results showed that transmigration capability of mDCs was markedly diminished after treatment with 50 ng/mL VEGF (Figure 3). Previous studies have also suggested that the TME and TGF-β_1_ cytokine can lead to improper migration of DCs [20,21,29,34]. That also further confirmed that the decreased EPM, hypo-osmolality resistance, and deformability of DCs can adversely affect their motility and may be one of the explanations for why only a few tumor-stimulated DCs can migrate to the lymph nodes after intracutaneous injection [41].

### 2.5. Analyses of Apoptosis and Specificity of the Effect of VEGF

**Figure 4:**
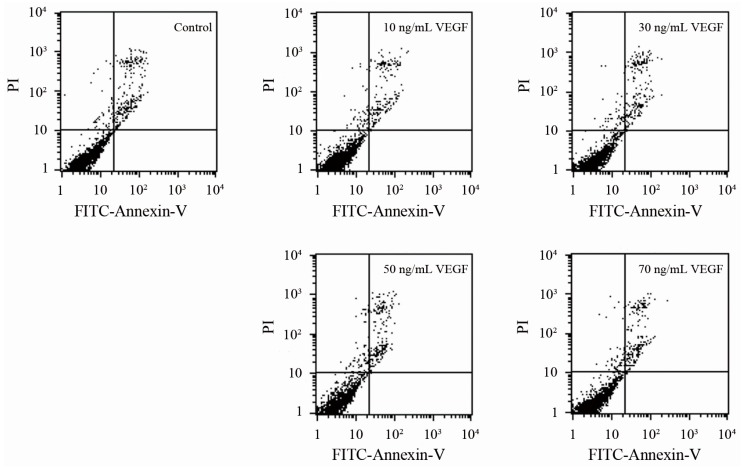
Apoptosis analysis of mDCs treated with different concentrations of VEGF.

**Figure 5:**
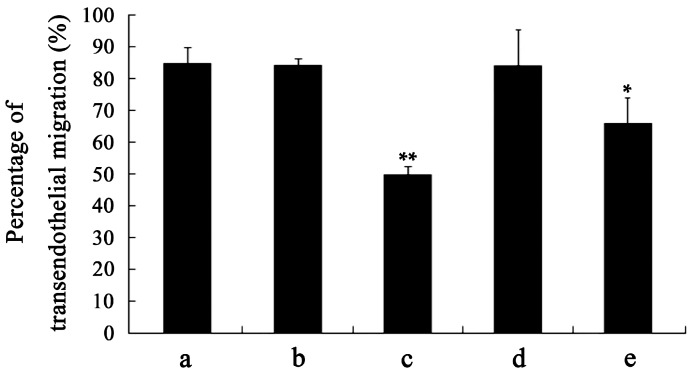
Specificity analysis of the effect of VEGF on mDCs. (**a**) control; (**b**) 50 ng/mL IgG; (**c**) 50 ng/mL VEGF; (**d**) 5 ng/mL TGF-β_1_; (**e**) 1 ng/mL IL-10. Compared with control group, * *p* < 0.05, ** *p* < 0.01.

To confirm whether the changes in biophysical properties of VEGF-treated DCs and the less tumor-stimulated DCs transmigrated to the lymph nodes were induced by their viability, an apoptosis analysis was performed. The results showed that there is no significant difference between the VEGF-treated mDCs and the control (Figure 4), as observed by others [36], illustrating that VEGF has only a slight influence on cell survival. Thus, VEGF could cause changes in biophysical characteristics, impairment of motility, and dysfunction of immune function of mDCs by mechanisms other than cell apoptosis. Additionally, the specificity of the effect of VEGF on mDCs was analyzed by comparing the influence of IgG, VEGF, TGF-β_1_, and IL-10 on the capacity of transendothelial migration. The result showed that the effect of VEGF on mDCs is specific relative to the IgG negative control, while that is just different to other cytokines (Figure 5); that also proved that it is equally important to study the effects of both single cytokines and a mixture.

### 2.6. Effects of VEGF on F-Actin Cytoskeleton

**Figure 6:**
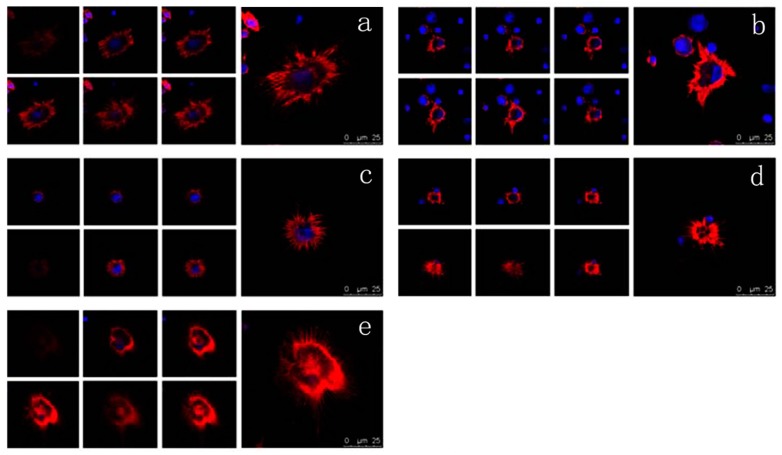
Confocal microscope analysis of F-actin organization of mDCs treated with different concentrations of VEGF (×600). (**a**) control; (**b**) 10 ng/mL; (**c**) 30 ng/mL; (**d**) 50 ng/mL; (**e**) 70 ng/mL.

**Figure 7:**
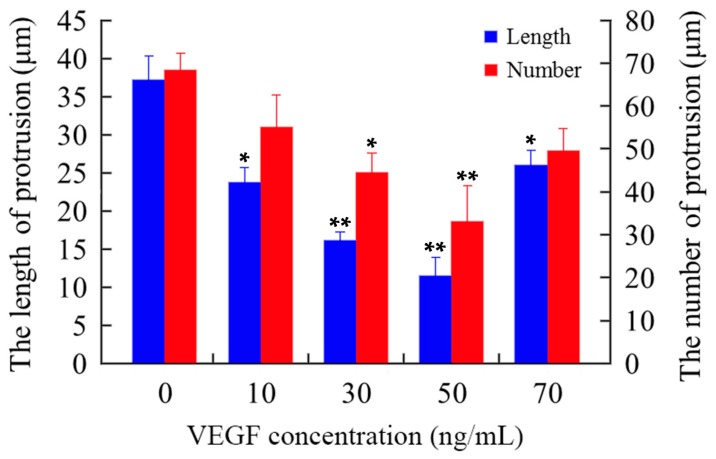
The filopodia on the surface of mDCs treated with different concentrations of VEGF. Cells were incubated with rhodamine phalloidin and photographed by confocal microscope. The blue columns represent the length of the filopodia, while the red columns represent the density of the filopodia. Compared with control group, * *p* < 0.05, ** *p* < 0.01.

The cytoskeleton is the basis of the structure and function of cells, and continuously regulates their viscoelasticity, deformability, motility, and signaling, especially F-actin organization [28,48,49,50,51]. Rhodamine phalloidin can specifically bind to F-actin in cells, but does not combine with G-actin, which can be used for analyzing the structure of F-actin cytoskeleton by determination of the rhodamine fluorescence using confocal microscope. As shown in Figure 6, the F-actin of untreated mDCs attached to the internal surface of the cell membrane and formed regular circles (Figure 6a). After treatment with different concentrations of VEGF, however, the F-actin of mDCs became irregular (Figure 6b–e), suggesting that their F-actin cytoskeleton was reorganized in response to VEGF. Moreover, the filopodia on the surface of mDCs were accordingly decreased after the cells were cultured in media containing different concentrations of VEGF (Figure 7). Similarly, 50 ng/mL VEGF had the greatest influence on the cytoskeleton of mDCs. The cytoskeletal changes or actin organization of both DCs and T cells are very important and necessary to form and maintain the structure of immune synapses and, probably, signaling from this region. Aberrant alterations of cytoskeleton may induce the reduction in the stimulatory ability of mDCs [44]. Therefore, it could be speculated that VEGF-induced cytoskeleton reorganization of mDCs can impair their motility and hamper the formation of immune synapses between mDCs and naive T cells.

### 2.7. Stimulatory Capability of mDCs

**Figure 8:**
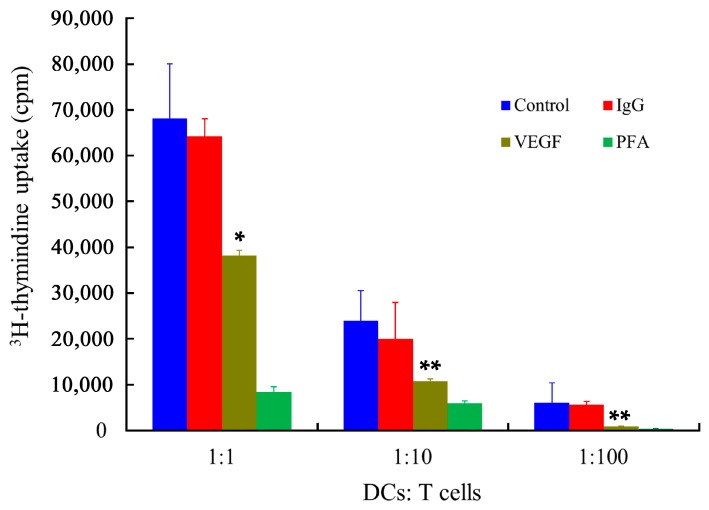
Effect of VEGF on the immune stimulatory capability of mDCs, as determined by MLR assay. The *x*-axis represents the ratio of cell numbers of mDCs to T cells. The *y*-axis represents the ^3^H-thymidine radioactivity counts per minute in a MicroBeta TRILUX liquid scintillation counter. Compared with control group, * *p* < 0.05, ** *p* < 0.01.

The main function of mDCs is their stimulatory capabilities, which were determined by the primary allogeneic mixed leukocyte reaction (MLR) assay, using human T lymphocytes as responder cells. As shown in Figure 8, the immune stimulatory capabilities of mDCs were dramatically affected by VEGF. In addition, Tourkova et al. have demonstrated that the biophysical characteristics of cells can be affected by a low concentration of paraformaldehyde (PFA), while the expression of cell surface markers is not remarkable different [52]. The results showed that the PFA-treated DCs almost totally lost their stimulatory ability, which provides direct support for the notion that biophysical properties, especially the deformability of mDCs, could be related to the regulation of antigen presentation and activation of T cells proliferation via different pathways. Despite tumor cells being able to downregulate some co-stimulatory molecules [20], it has been demonstrated that the phenotype of DCs remains unchanged regardless of VEGF treatment, while VEGF-treated DCs are less able to stimulate antigen-specific T cells [36,37]. Therefore, these results confirmed that the biophysical characteristics and the expression of molecular markers of mDCs are both vitally important for antigen presentation and T cells activation [53].

### 2.8. Differentially Expressed Genes of VEGF-Treated mDCs

**Figure 9:**
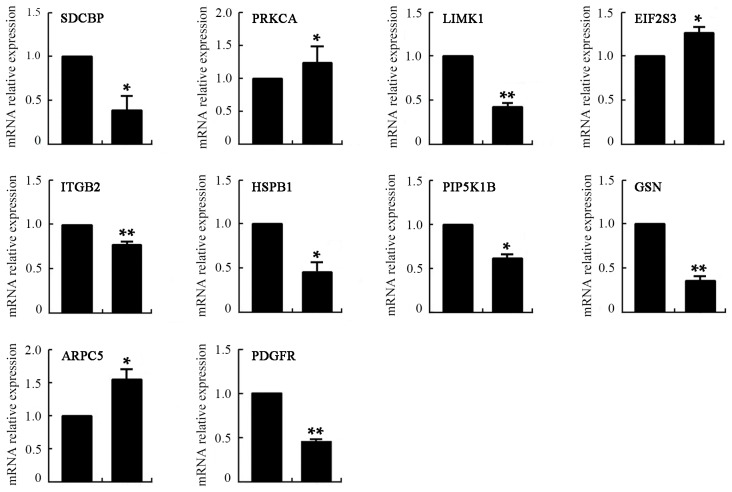
The mRNA expression levels of mDCs after treatment with 50 ng/mL VEGF determined by real-time PCR. Compared with control group, * *p* < 0.05, ** *p* < 0.01.

In a microarray analysis, gene expression profiles were considered to be different when a two-fold change was reached. The results showed that 2445 genes and 2417 genes were up- and downregulated, respectively, after treatment with 50 ng/mL VEGF. Gene Ontology (GO) term analysis illustrated that these genes were related to cellular functions, such as immune response, cell migration, the structural constitution of cytoskeleton, and so on (Table 2 and Table 3). Furthermore, Kyoto Encyclopedia of Genes and Genomes (KEGG) analysis was carried out to analyze the relevant signaling pathway of the differentially expressed genes. The results showed that a total of 140 signaling pathways were correlated with differentially expressed genes, from which 11 pathways (including MAPK, T cell receptor, leucocyte migration, cytoskeleton reorganization, cell adhesion, adhesion molecules, and antigen process and presentation) are associated with the most differentially expressed genes (Table 4). The expression of 10 genes correlated with the cytoskeleton rearrangement and motility of cells was further validated by quantitative real-time PCR (Figure 9). The mRNA expression levels of SDCBP, LIMK1, ITGB2, HSPB1, PIP5K1B, GSN, and PDGFR were downregulated, while those of PRKCA, EIF2S3 and ARPC5 were upregulated (* *p* < 0.05 or ** *p* < 0.01) after treatment with 50 ng/mL VEGF. Relative to the results of the microarray analysis, PIP5K1B and ARPC5 were not identical in real-time PCR detection. Thus, approximately 80% of the genes were consistent between the analysis results of microarray and real-time PCR. Thus, VEGF can regulate the expression of numerous genes, indicating that it has great influence on the function of DCs. The proteins encoded by some of the up- or downregulated genes, such as SDCBP, LIMK1, ITGB2, PIP5K1B, GSN, and ARPC5, can affect actin depolymerization, filopodia formation, adhesion, and cell migration. As a result, changes in these genes induced by VEGF may lead to abnormal reorganization of the F-actin cytoskeleton and deterioration of deformability and/or motility, and subsequently cause dysfunction of the immune function of mDCs. Determining the underlying molecular mechanisms will be our ongoing goal.

## 3. Materials and Methods

### 3.1. Isolation of Monocytes and Generation of DCs

DCs were prepared from fresh peripheral blood mononuclear cells (PBMCs) of healthy donors as described [21]. The donors gave informed consent to this experimental study, approved by the ethics committee of Guizhou Medical University. Highly enriched CD14^+^ monocytes were isolated from peripheral blood by Ficoll-Paque gradient centrifugation and purified by cocktail immunomagnetic beads (Dynal, Oslo, Norway). The monocytes were cultured in RPMI 1640 supplemented with 14% fetal bovine serum (FBS) (Gibco, Waltham, MA, USA), 1% penicillin/streptomycin, 150 ng/mL recombinant human GM-CSF (rhGM-CSF), and 100 ng/mL recombinant human IL-4 (rhIL-4) (Peprotech, Rocky Hill, NJ, USA). On day 7, 10 ng/mL recombinant human TNF-α (rhTNF-α) (Peprotech, Rocky Hill, NJ, USA) was added for another 72 h culture. The phenotypes of DCs were analyzed using flow cytometer (FACScan, Becton Dickinson, San Jose, CA, USA) by staining the cell surface with FITC- or PE-conjugated mouse anti-human CD11c, CD40, CD80, CD83, CD86, CCR7, and HLA-DR (Sigma-Aldrich, St. Louis, MO, USA). The trypan blue staining was applied to analyze the viability of cells as described [54].

### 3.2. Culture of HY926 Cell Lines

The human umbilical vein endothelial cells (HUVEC) line HY926 was a gift from Dr. Yi Song of the School of Public Health, Peking University. HY926 cells were cultured in a DMEM medium supplemented with 10% FBS and incubated in 5% CO_2_ incubator at 37 °C.

### 3.3. Treatment of VEGF on mDCs

Based on other studies [35,36,37,38] and our preliminary experiments, concentrations of 0, 10, 30, 50, and 70 ng/mL was set up and used to co-culture mDCs. The different concentrations of recombinant human VEGF_165_, TGF-β_1_, or IL-10 (Peprotech, Rocky Hill, NJ, USA) were added into dishes containing 1 × 10^6^ mDCs. After 24 h culture, the cells were collected for further analysis.

### 3.4. Measurement of Cell EPM

EPM was measured as previously described [21]. Briefly, mDCs were cultured and adjusted to 2 × 10^6^ cells/mL with 9% saccharose solution. The cell electrophoresis meter (LIANG-100, Shanghai Medical University, Shanghai, China) was used to examine the EPM with the voltage of 40 V at 30 °C. Ten cells were randomly selected for each sample and three repeated experiments were performed for standard deviation (SD) analyses.

### 3.5. Measurement of Cell Osmotic Fragility

Solutions with different osmotic concentrations (0, 25, 55, 85, 115, 145, 175, 205, 235, 265, and 295 mOsm/kg) were prepared by mixing PBS with distilled water in various proportions. Eleven aliquots of the cell suspensions (2 × 10^6^ cells/mL) were separately pipetted into Eppendorf tubes and then centrifuged at 800× *g* for 8 min. Three hundred microliters of solutions with different osmotic concentrations was added to resuspend cells. After 30 min, the numbers of nonhemolyzed cells were counted using a hemocytometer. The percentage of nonhemolyzed cells relative to that in the osmotic concentration of 295 mOsm/kg was analyzed [21].

### 3.6. Micropipette Measurement of Cell Viscoelasticity

Micropipette measurements were performed as previously described [27,28]. Briefly, 50 μL of cell suspension (1 × 10^6^ cells/mL) were added into a chamber located on microscope stage. By micromanipulation, the micropipette tip was positioned near a single cell and negative pressure was exerted to aspirate a fraction of the cell into the micropipette. The time course of cell deformation was continuously recorded using a video recorder. Sequential photographs were obtained from the recorded images during single-frame replay on the video monitor every 80 ms. The length of the cell tongue aspirated into the micropipette was measured.

### 3.7. Measurement of mDCs Transmigration in Transwell

In Transwell chamber, mDCs (1 × 10^6^ cells) treated with or without cytokines were pipetted into the upper compartment that was covered with a HUVEC monolayer, while an RPMI 1640 medium supplemented with 14% FBS and 0.6 mg/mL chemokine CCL19 (R & D, McKinley Place NE, Minneapolis, MN, USA) were added to the lower compartment. After being cultured at 37 °C for 12 h in 5% CO_2_ incubator, DCs in the lower compartment were collected and the numbers were counted with a FACScan (Becton Dickinson, Mountain View, San Jose, CA, USA) as previously described [29]. The ratio of the counted numbers to the initial adding amount represents the percentage of transendothelial migration.

### 3.8. Cell Apoptosis Analysis

Annexin V-FITC/PI distaining kit was used to analyze the cell apoptosis. Five microliters of Annexin V-FITC (Qihai, Shanghai, China) were pipetted into 400 µL mDCs suspension (1 × 10^6^ cells/mL) and incubated for 15 min at 4 °C in the dark. Then, 10 µL PI were added and incubated for 5 min in the same conditions. The cells were analyzed using a flow cytometer and Cell Quest Software (Becton Dickinson, San Jose, CA, USA).

### 3.9. Confocal Laser Scanning Microscopy (CLSM) Analysis

DCs were immobilized in cover slips with poly-l-lysine and fixed using PFA. After washing with 100 mmol/L glysine and PBS buffer, 500 µL PBS containing 1% bovine serum albumin was used for blocking. Then, 500 µL PBS and 10 µL rhodamine phalloidin (Invitrogen, Waltham, MA, USA) and DAPI were added and stationarily incubated for 30 min at room temperature in the dark. The cover slips were transferred with the embedded DCs on them by adding Antifade Mounting Medium (Beyotime, Haimen, China) and detected using CLSM (Leica Lasertechnik, Heidelberg, Germany). Three-dimensional images were reconstructed using Leica software.

### 3.10. Mixed Leukocyte Reaction (MLR)

As positive and negative controls, mDCs were fixed with 1% PFA for 10 min at 4 °C and 50 ng/mL IgG for 24 h prior to assay. Allogeneic T cells were obtained from a fresh PBMC suspension by passage through the nylon wool columns after the removing of platelets with PBS. MLR assays were carried out in round-bottomed 96-well plates to ensure efficient contact between DCs and T cells. The cells were separately added in triplicate in graded doses (10^5^, 10^4^, 10^3^ cells/well) to T cells (1 × 10^5^ cells/well) in a total volume of 200 μL of the RPMI 1640 medium supplemented with 10% FBS. Proliferation of T cells was measured from the uptake of ^3^H-thymidine (1 μCi/well, 5 μCi/mmol, DuPont-NEN, Boston, MA, USA). The culture was harvested on GF/C glass fiber filter paper (Whatman, Maidstone, UK) using a MACH III microwell harvester (Tomtec, Hamden, CT, USA). Incorporation of ^3^H-thymidine was determined from the radioactivity counts per minute in a MicroBeta TRILUX liquid scintillation counter (Wallac, Gaithersburg, MD, USA).

### 3.11. Microarray and Real-Time PCR Analyses

Total RNAs of DCs treated with or without 50 ng/mL VEGF were isolated and gene expression profiles were analyzed by CapitalBio Corporation (Beijing, China). The data were analyzed using CapitalBio Molecule Annotation System (MAS 3.0) and the genes with more than two-fold changes were considered as differentially expressed genes. The expression level of some genes was further validated by real-time PCR analysis using specific primers (Table 5).

### 3.12. Statistical Analysis

All experiments were performed at least in triplicate under the same conditions and the data were analyzed by the analysis of variance (ANOVA two-way) of SPSS-statistics-v17.0. Student’s *t*-tests were carried out and differences were considered statistically significant at *p* < 0.05 and highly significant at *p* < 0.01.

## 4. Conclusions

The current study characterized the effects of recombinant human VEGF on the biophysical characteristics and motility of DCs from an interdisciplinary viewpoint. Firstly, the mDCs were treated with different concentrations of VEGF and their changes of biophysical properties, including EPM, osmotic fragility, deformability, and transmigration were analyzed. The results showed that VEGF had a negative effect on these biophysical properties of mDCs; 50 ng/mL VEGF had the greatest influence. Then, the viability of VEGF-treated mDCs was determined, excluding those effects induced by their apoptosis. Subsequently, the F-actin cytoskeleton and filopodia of VEGF-treated mDCs were analyzed by confocal microscope. The results showed that VEGF could induce actin polymerization, i.e., cytoskeleton reorganization, supporting the view that the biophysical properties and motility of mDCs were deteriorated by VEGF through cytoskeleton remodeling. The MLR assay further confirmed that the main immune function of mDCs for stimulating T lymphocyte proliferation was dramatically affected by VEGF. In the end, the differentially expressed genes of VEGF-treated mDCs were identified by microarray analysis and 10 genes associated with the cytoskeleton rearrangement and motility of cells were further verified by real-time PCR. A total of 2445 and 2417 genes were up- and downregulated, respectively, after treatment with 50 ng/mL VEGF. These genes were correlated to cellular functions, such as immune response, cell migration, cell adhesion, and regulation of actin cytoskeleton. Moreover, approximately 80% of genes were consistent between the analysis results of microarray analysis and real-time PCR detection. In summary, VEGF at high concentrations could impair the biophysical properties and motility of mDCs by remodeling the cytoskeleton of cells.

## Figures and Tables

**Table 1:** Electrophoretic mobility of mDCs treated with different concentrations of VEGF.

Concentration of VEGF (ng/mL)	0	10	30	50	70
EPM	3.063 ± 0.292	2.775 ± 0.265	0.801 ± 0.200 *	0.729 ± 0.108 **	1.801 ± 0.185

Compared with control group, * *p* < 0.05, ** *p* < 0.01. Values represent mean ± SD.

**Table 2:** GO term analysis of the upregulated genes.

GO Term	Count	*p*-Value	*q*-Value
GO:0003777 microtubule motor activity	16	9.23 × 10^−23^	1.59 × 10^−21^
GO:0003779 actin binding	16	7.38 × 10^−13^	7.46 × 10^−12^
GO:0051015 actin filament binding	6	2.15 × 10^−8^	1.32 × 10^−7^
GO:0005200 structural constituent of cytoskeleton	6	7.26 × 10^−7^	3.56 × 10^−6^
GO:0008017 microtubule binding	5	2.59 × 10^−6^	1.12 × 10^−5^
GO:0051059 NF-kappaB binding	4	3.08 × 10^−6^	1.32 × 10^−5^
GO:0005089 Rho guanyl-nucleotide exchange factor activity	5	1.44 × 10^−5^	5.31 × 10^−5^
GO:0015631 tubulin binding	4	2.28 × 10^−4^	5.67 × 10^−4^
GO:0050431 transforming growth factor beta binding	2	7.75 × 10^−4^	0.00159
GO:0003774 motor activity	4	0.003258	0.005246
GO:0030616 transforming growth factor beta receptor, common-partner cytoplasmic mediator activity	1	0.004199	0.005246
GO:0070123 transforming growth factor beta receptor activity, type III	1	0.004199	0.005246
GO:0050839 cell adhesion molecule binding	2	0.004958	0.006071
GO:0005100 Rho GTPase activator activity	2	0.005356	0.006509
GO:0046332 SMAD binding	2	0.010646	0.010604
GO:0005522 profilin binding	1	0.02082	0.016575
GO:0030898 actin-dependent ATPase activity	1	0.024932	0.01898
GO:0030675 Rac GTPase activator activity	1	0.033104	0.023774
GO:0005072 transforming growth factor beta receptor, cytoplasmic mediator activity	1	0.045234	0.030536
GO:0042288 MHC class I protein binding	1	0.053235	0.034859
GO:0008093 cytoskeletal adaptor activity	1	0.053235	0.034859
GO:0000146 microfilament motor activity	1	0.06117	0.039009
GO:0042287 MHC protein binding	1	0.096063	0.056973
GO:0017048 Rho GTPase binding	1	0.107403	0.062869
GO:0008092 cytoskeletal protein binding	3	0.299076	0.160163

**Table 3:** GO term analysis of the downregulated genes.

GO Term	Count	*p*-Value	*q*-Value
GO:0003779 actin binding	39	1.09 × 10^−44^	4.76 × 10^−43^
GO:0046332 SMAD binding	9	3.39 × 10^−14^	4.02 × 10^−13^
GO:0032395 MHC class II receptor activity	6	1.32 × 10^−11^	1.24 × 10^−10^
GO:0034713 type I transforming growth factor beta receptor binding	4	9.52 × 10^−9^	6.15 × 10^−8^
GO:0051015 actin filament binding	6	1.80 × 10^−8^	1.12 × 10^−7^
GO:0005200 structural constituent of cytoskeleton	6	6.11 × 10^−7^	3.03 × 10^−6^
GO:0003774 motor activity	7	2.37 × 10^−6^	9.99 × 10^−6^
GO:0005021 vascular endothelial growth factor receptor activity	3	3.72 × 10^−6^	1.53 × 10^−5^
GO:0050431 transforming growth factor beta binding	3	7.93 × 10^−6^	3.04 × 10^−5^
GO:0048365 Rac GTPase binding	3	7.93 × 10^−6^	3.04 × 10^−5^
GO:0030617 transforming growth factor beta receptor, inhibitory cytoplasmic mediator activity	2	1.66 × 10^−5^	5.64 × 10^−5^
GO:0042289 MHC class II protein binding	2	4.96 × 10^−5^	1.51 × 10^−4^
GO:0003823 antigen binding	6	8.13 × 10^−5^	2.34 × 10^−4^
GO:0050839 cell adhesion molecule binding	3	1.45 × 10^−4^	3.87 × 10^−4^
GO:0017048 Rho GTPase binding	3	1.84 × 10^−4^	4.57 × 10^−4^
GO:0030274 LIM domain binding	2	2.46 × 10^−4^	5.91 × 10^−4^
GO:0008017 microtubule binding	3	0.001215	0.002335
GO:0045296 cadherin binding	2	0.001256	0.002393
GO:0008093 cytoskeletal adaptor activity	2	0.001256	0.002393
GO:0000146 microfilament motor activity	2	0.001682	0.003063
GO:0043183 vascular endothelial growth factor receptor 1 binding	1	0.004075	0.005707
GO:0043184 vascular endothelial growth factor receptor 2 binding	1	0.004075	0.005707
GO:0051059 NF-kappaB binding	2	0.004315	0.005707
GO:0005100 Rho GTPase activator activity	2	0.005054	0.006579
GO:0004920 interleukin-10 receptor activity	1	0.008134	0.00876
GO:0042805 actinin binding	1	0.016201	0.014943
GO:0005522 profilin binding	1	0.020211	0.017634
GO:0030618 transforming growth factor beta receptor, pathway-specific cytoplasmic mediator activity	1	0.020211	0.017634
GO:0005025 transforming growth factor beta receptor activity, type I	1	0.024204	0.019893
GO:0030675 Rac GTPase activator activity	1	0.032141	0.025015
GO:0003777 microtubule motor activity	2	0.039709	0.029659
GO:0005072 transforming growth factor beta receptor, cytoplasmic mediator activity	1	0.043925	0.032045
GO:0005024 transforming growth factor beta receptor activity	1	0.063249	0.043469
GO:0042287 MHC protein binding	1	0.093358	0.06095
GO:0008092 cytoskeletal protein binding	4	0.117053	0.074554

**Table 4:** KEGG pathway analysis of the differentially expressed genes.

Pathway	Up-Regulated Genes			Down-Regulated Genes		
	Count	*p*-Value	*q*-Value	Count	*p*-Value	*q*-Value
T cell receptor signaling pathway	19	1.07 × 10^−12^	5.63 × 10^−12^	5	0.096195	0.026281
Leukocyte transendothelial migration	13	1.39 × 10^−6^	2.56 × 10^−6^	17	1.75 × 10^−9^	2.65 × 10^−9^
Regulation of actin cytoskeleton	17	3.30 × 10^−6^	5.31 × 10^−6^	27	6.92 × 10^−13^	1.86 × 10^−12^
Cell adhesion molecules (CAMs)	13	4.40 × 10^−6^	6.97 × 10^−6^	34	3.15 × 10^−26^	4.09 × 10^−25^
Focal adhesion	15	2.60 × 10^−5^	3.36 × 10^−5^	32	5.62 × 10^−18^	2.83 × 10^−17^
Primary immunodeficiency	6	7.30 × 10^−5^	8.11 × 10^−5^	7	1.02 × 10^−5^	7.72 × 10^−6^
Antigen processing and presentation	8	5.09 × 10^−4^	4.63 × 10^−4^	21	4.90 × 10^−16^	2.12 × 10^−15^
B cell receptor signaling pathway	7	9.03 × 10^−4^	7.70 × 10^−4^	14	1.05 × 10^−9^	1.65 × 10^−9^
VEGF signaling pathway	5	0.020369	0.011837	8	2.87 × 10^−4^	1.55 × 10^−4^
TGF-beta signaling pathway	5	0.033979	0.018626	11	3.72 × 10^−6^	3.10 × 10^−6^
MAPK signaling pathway	5	0.54 × 10^−4^	0.81 × 10^−4^	33	4.95 × 10^−15^	1.98 × 10^−14^

**Table 5:** Primers used in real-time PCR analysis.

Gene	Primers	PCR Product (bp)	T_m_ (°C)
*SDCBP*	5′-GGGTTTGCAGAAAAAGCAGT-3′	94	58
	5′-GAGCGGTTCCTTGTGGG-3′		
*PRKCA*	5′-CAAATTCATGGCACCTCTTG-3′	91	59
	5′-CGAGGTGAAGGACCACAAAT-3′		
*LIMK1*	5′-TAGTACTGGTGCGACAGGGA-3′	105	60
	5′-GGAGAGGAAGGAAGCGAGTT-3′		
*EIF2S3*	5′-TGGCTTGATTGTCACTCCTC-3′	109	60
	5′-TTTTGACCAATCCAGTGTGC-3′		
*ITGB2*	5′-TGCTGACCTTGAACTTCGTG-3′	108	60
	5′-GGACTCCAGCACACCGAG-3′		
*HSPB1*	5′-GTGAAGCACCGGGAGATGTA-3′	113	60
	5′-AGCTGACGGTCAAGACCAAG-3′		
*PIP5K1B*	5′-AGAAAAGGCCCTCAAATGCT-3′	100	59
	5′-GTGAGATGAGGAAGCCAAGG-3′		
*GSN*	5′-CTTCCCTGCCTTGAGGAACT-3′	108	60
	5′-CAGCAGCAGGTAGTGCTCAT-3′		
*ARPC5*	5′-CCACTTCCTGCCAGACTACAC-3′	100	60
	5′-GCCCGTCTGACAATAGCAGT-3′		
*PDGFR*	5′-CGAATGGTCACCCGAGTTT-3′	105	61
	5′-CTTTAAGAAGGCCACGGTGA-3′		
